# Supratotal Resection of Gliomas With Awake Brain Mapping: Maximal Tumor Resection Preserving Motor, Language, and Neurocognitive Functions

**DOI:** 10.3389/fneur.2022.874826

**Published:** 2022-05-12

**Authors:** Kazuya Motomura, Fumiharu Ohka, Kosuke Aoki, Ryuta Saito

**Affiliations:** Department of Neurosurgery, Nagoya University School of Medicine, Nagoya, Japan

**Keywords:** gliomas, overall survival, progression-free survival, extent of resection, subcortical fiber, supratotal resection, awake brain mapping

## Abstract

Gliomas are a category of infiltrating glial neoplasms that are often located within or near the eloquent areas involved in motor, language, and neurocognitive functions. Surgical resection being the first-line treatment for gliomas, plays a crucial role in patient outcome. The role of the extent of resection (EOR) was evaluated, and we reported significant correlations between a higher EOR and better clinical prognosis of gliomas. However, recurrence is inevitable, even after aggressive tumor removal. Thus, efforts have been made to achieve extended tumor resection beyond contrast-enhanced mass lesions in magnetic resonance imaging (MRI)-defined areas, a process known as supratotal resection. Since it has been reported that tumor cells invade beyond regions visible as abnormal areas on MRI, imaging underestimates the true spatial extent of tumors. Furthermore, tumor cells have the potential to spread 10–20 mm away from the MRI-verified tumor boundary. The primary goal of supratotal resection is to maximize EOR and prolong the progression-free and overall survival of patients with gliomas. The available data, as well as our own work, clearly show that supratotal resection of gliomas is a feasible technique that has improved with the aid of awake functional mapping using intraoperative direct electrical stimulation. Awake brain mapping has enabled neurosurgeons achieve supratotal resection with favorable motor, language, and neurocognitive outcomes, ensuring a better quality of life in patients with gliomas.

## Introduction

Gliomas are a heterogeneous population of intrinsic brain tumors of the central nervous system (1–4). Among malignant histological subtypes such as glioblastoma [GBM; World Health Organization (WHO) grade IV], and WHO grade II and III gliomas, GBM is the most aggressive and common form of malignant primary brain tumor. The clinical prognosis of GBM is poor and the median overall survival (OS) is only 12–18 months after diagnosis, even with intensive treatments such as surgery and chemoradiotherapy (5, 6). Surgical intervention plays a crucial role in the treatment of GBM; tumors invariably recur even after aggressive tumor resection and most patients eventually succumb to the disease. The slow-growing primary brain tumors (1, 2), WHO grade II and III gliomas (hereon, referred to as, lower-grade gliomas) are diagnosed based on molecular features (presence or absence of *IDH*1/2 mutations and 1p/19q co-deletion) (1, 2). Over time, lower-grade gliomas typically undergo malignant transformation to GBMs, and the median survival is only 7.8–31 months, even with intensive treatment including surgery, chemotherapy, and radiotherapy (7, 8).

Several studies have reported a significant relationship between the extent of resection (EOR) in patients with gliomas and survival rate (3, 4, 9–14). Recent studies also provided evidence that increased EOR and decreased postsurgical tumor volume are related to increased progression-free survival (PFS) and OS in diffuse gliomas (4, 14, 15). Therefore, quantifying the EOR is of particular interest for patients with diffuse gliomas. To achieve increased survival rates, an increase in the EOR of tumors has been achieved using modalities such as ultrasound-guided resection (16), neuro-navigation (16), intraoperative magnetic resonance imaging (iMRI) (17, 18), and 5-aminolevulinic acid (5-ALA) (19). Furthermore, Duffau et al. reported that aggressive resection of low-grade gliomas using intraoperative awake mapping to identify functional areas in the brain, can result in supratotal resection and improved patient survival (20–22).

Here, we present a systematic review of the literature on supratotal resection in patients with gliomas with the aim of evaluating the survival benefit of supratotal resection for diffuse gliomas. In particular, we focused on the effects of supratotal resection on glioma survival outcomes.

## Methods

We performed an extensive systematic literature review of the PubMed, Web of Science, and Scopus databases. We used the following keywords: “supratotal resection,” “supramarginal resection,” “awake brain mapping” for searching original articles, meta-analyses, reviews, clinical series, and case reports.

## Results

**Figure 1:**
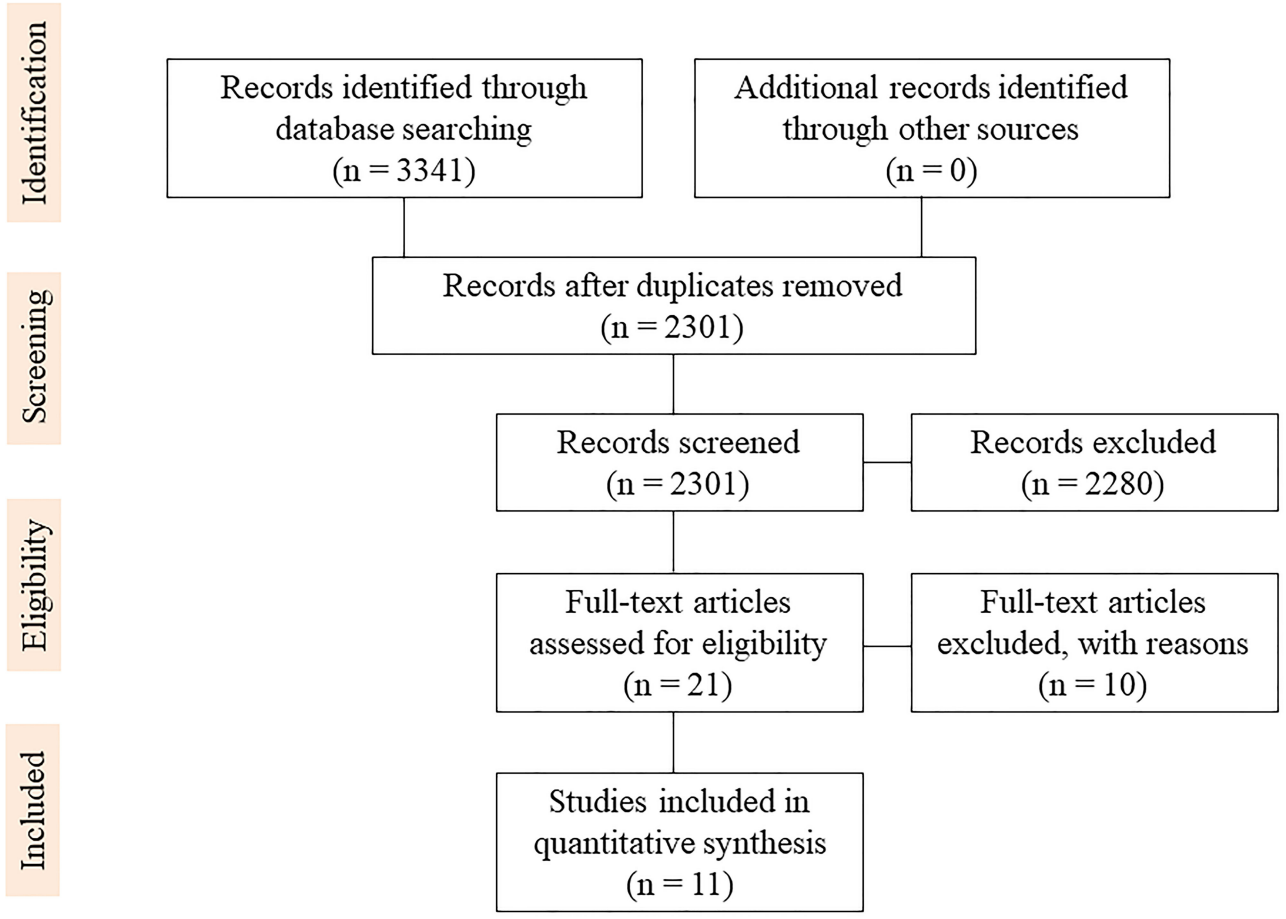
PRISMA flow diagram.

The literature search yielded 3,341 studies regarding supratotal resection using awake brain mapping. Duplicates were removed from 2301 studies. In total, 21 full texts were reviewed for eligibility. Eleven clinical studies were included in the final review, as shown in the PRISMA flowchart (23) (Figure 1).

## Discussion

### Glioblastomas and EOR

Several studies evaluating the EOR for GBM have shown that maximal tumor removal is significantly associated with better clinical outcomes (3, 24). The correlation between EOR and improved survival is widely agreed upon in the field of surgical neuro-oncology. Many retrospective cohort studies have revealed that increased EOR, ranging from 78 to 98%, improves OS in patients with newly diagnosed GBM (3, 25). Lacroix et al. analyzed 416 consecutive patients with GBM who underwent tumor resection and reported that tumor resection of 98% or more was associated with a survival advantage (25). While they used the outcome scale based on age, Karnofsky Performance Scale (KPS) score, and tumor necrosis on MRI, they concluded that patients with lower scores who underwent aggressive resections were associated with significantly longer survival. Multivariate analysis using a Cox proportional hazards model demonstrated that age (<45, 45–64, >64 years), performance score (<80), necrosis, enhancement, and EOR (≥98%) were significantly related to the survival of GBM patients. Stummer et al. performed a randomized phase III trial of 270 newly diagnosed patients with GBM to evaluate the effect of fluorescence-guided resection with 5-aminolevulinic acid (5-ALA) on clinical outcomes (26). A total of 139 patients were assigned to surgical resection with the fluorescent adjunct 5-ALA and 131 patients were assigned to the white-light control group. The clinical outcome revealed that complete resection was achieved in 65% of 5-ALA cases and 36% of white-light cases. Patients treated with 5-ALA had significantly higher 6-month PFS (41.0%) than in those treated with white light (21.1%).

### WHO Grade II Gliomas and EOR

Some studies investigating the role of EOR in gliomas have revealed that extensive surgical resection favors longer survival in WHO grade II gliomas (9–13, 24, 27). A large cohort study including 216 patients with WHO grade II gliomas revealed that patients with an EOR > 90% had 5- and 8-year OS rates of 97 and 91%, respectively, whereas those with an EOR <90% had 5- and 8-year OS rates of 76 and 60%, respectively (9). Another study in Norway examined survival in population-based cohorts of WHO grade II gliomas from two university hospitals by distinct surgical treatment strategies. Early surgical resection was performed at one university hospital, whereas biopsy and watchful waiting were preferred for WHO grade II gliomas at the other hospital. Notably, this study revealed 5-year OS rates of 60% and 74% in patients in the “biopsy and observation” and “early surgical resection” groups, respectively (12, 13). The survival data of patients treated with biopsy showed significantly better OS than for those treated with early surgical resection. Moreover, another research group reported a significant survival benefit for patients who underwent “early tumor resection” compared to those who underwent only “biopsy” (5-year OS: 82 vs. 54%) at two distinct departments acting independently at the same university medical center (27). These findings suggest that glioma neurosurgeons should attempt to perform maximal safe tumor resection by increasing the EOR to prolong survival in patients with WHO grade II gliomas.

### WHO Grade III Gliomas and EOR

Several studies on WHO grade III gliomas have shown that extensive tumor resection affects the OS of patients (28–30). The EOR of tumors had been reported to be significantly related to OS in WHO grade III anaplastic oligodendroglial tumors in a European phase III clinical trial (EORTC-26951) (28). Nomiya et al. evaluated the prognostic parameters of 170 patients with WHO grade III anaplastic astrocytoma (30). Patients who achieved total or subtotal tumor resection showed a significantly more favorable clinical outcome with a median OS of 62.9 months than those patients who underwent partial resection or biopsy alone, with a median OS of 22.9 months. The authors emphasized that the EOR of tumors was the most powerful prognostic factor for the treatment of WHO grade III anaplastic astrocytomas in their cohort study. Kawaguchi et al. demonstrated the clinical importance of surgical tumor resection by investigating 124 consecutive patients with WHO grade III gliomas treated at a single institution (29). Among the group with *IDH*1/2 mutations and non-1p/19q co-deletions, survival was significantly longer in those with gross total removal than in those in whom gross total removal was not accomplished (median OS: not reached vs. 77 months). Therefore, extensive surgical tumor removal is essential to improve the prognosis of patients with WHO grade III gliomas, especially for astrocytic tumors with the *IDH*1/2 mutation lacking the 1p/19q co-deletion.

### WHO Grade II and III Gliomas and EOR

In this context, surgical tumor resection is considered the first-line treatment for WHO grade II and grade III gliomas. To date, several clinical studies have provided evidence supporting maximum safe tumor resection and early surgical intervention, which prolong survival in patients with WHO grade II and grade III gliomas (9, 11–13, 24, 27, 31, 32). From an ethical perspective, we could not perform randomized controlled trials examining the relationship between a higher EOR and better outcomes of WHO grade II and grade III gliomas, as extensive radiological tumor resection of WHO grade II and grade III gliomas is a better choice.

### Supratotal Resection and Awake Brain Mapping

MRI diffusion tractography (DT) is used to estimate subcortical fiber tracts before awake surgery. Voets et al. evaluated DT sensitivity, specificity, and accuracy for five subcortical fiber tracts, including the corticospinal tract (CST), arcuate fasciculus (AF), inferior fronto-occipital fasciculus (IFOF), optic radiation (OR), and inferior longitudinal fasciculus (ILF) (33). The sensitivity and specificity of preoperative DT predictions were very high, at 92.2 and 69.2%, respectively, and it varied across tracts. The authors concluded that preoperative DT of the navigation system is a reliable tool for accurately predicting the spatial location of subcortical fiber tracts in relation to a tumor during awake surgery. However, the information of some subcortical fiber tracts by DT cannot inform its neurological functions. Moreover, DT does not provide a critical surgical strategy directly during tumor resection in the operating room. Therefore, this technique is difficult to replace intraoperative direct cortical and subcortical stimulation during awake brain mapping.

Considering the infiltrative behavior of gliomas, the goal of maximal tumor removal should be balanced against the risk of neurological dysfunctions such as motor, language, and neurocognitive impairment (34, 35). Awake functional mapping has been proposed as the optimal strategy for patients with gliomas to accomplish maximal safe resection and preservation of neurological functions (36–40). Using awake surgical techniques with both cortical and subcortical functional mapping, a maximum degree of resection is possible while determining the functional boundaries (37, 41). Direct electrical stimulation in glioma patients triggers an increase in the EOR of tumors (42), an improvement in OS and PFS (43), and a significant decrease in the rate of postoperative permanent neurological deficits, including motor, language, and neurocognitive functions (38). When combined with intraoperative tasks for motor and language, awake mapping can detect possible anatomo-functional associations, allowing a deeper understanding of neuroconnectivity in the human brain. In particular, as surgery carries a high risk of injury to subcortical pathways, resulting in permanent neurological impairments, for which functional connectivity is a central limitation of neural regeneration, a better understanding of the connectome is essential for comprehending cortico-subcortical pathways and their relevance to tumor resection during awake surgery.

Although awake brain mapping represents the gold standard technique for identifying and preserving the eloquent areas, it must be acknowledged that awake surgery has some risks and complications. There are some problems regarding the risk of intraoperative seizures (44, 45), variability in anesthetic techniques (46, 47), difficulty in the selection and interpretation of intraoperative tasks (48, 49), and intraoperative pain and discomfort during the procedure (50, 51). In particular, the occurrence of intraoperative seizures is especially crucial, as it is related to procedure failure, reduction of the EOR, and a high incidence of postoperative motor and speech disturbance (52). Furthermore, the risk of intraoperative seizures has been reported to be higher in patients with intra-axial brain tumors during awake surgery (44). Therefore, the decision to perform awake mapping should be considered with caution.

In cases where functional boundaries are detected inside a tumor mass, surgical resection should involve subtotal or partial removal. When functional boundaries are observed outside the tumor mass, gross total or supratotal resection can be performed. Extended resection beyond the tumor margins of abnormalities in MRI-defined areas is termed supratotal resection (20, 53). Supratotal resection of GBM or WHO grade II or grade III gliomas is a newly developed concept that may improve the survival of patients with gliomas. Even with aggressive and intensive tumor surgery, awake brain mapping enables safe maximal resection of language-dominant or non-dominant tumors, preserving both the cortical and subcortical fibers.

**Figure 2:**
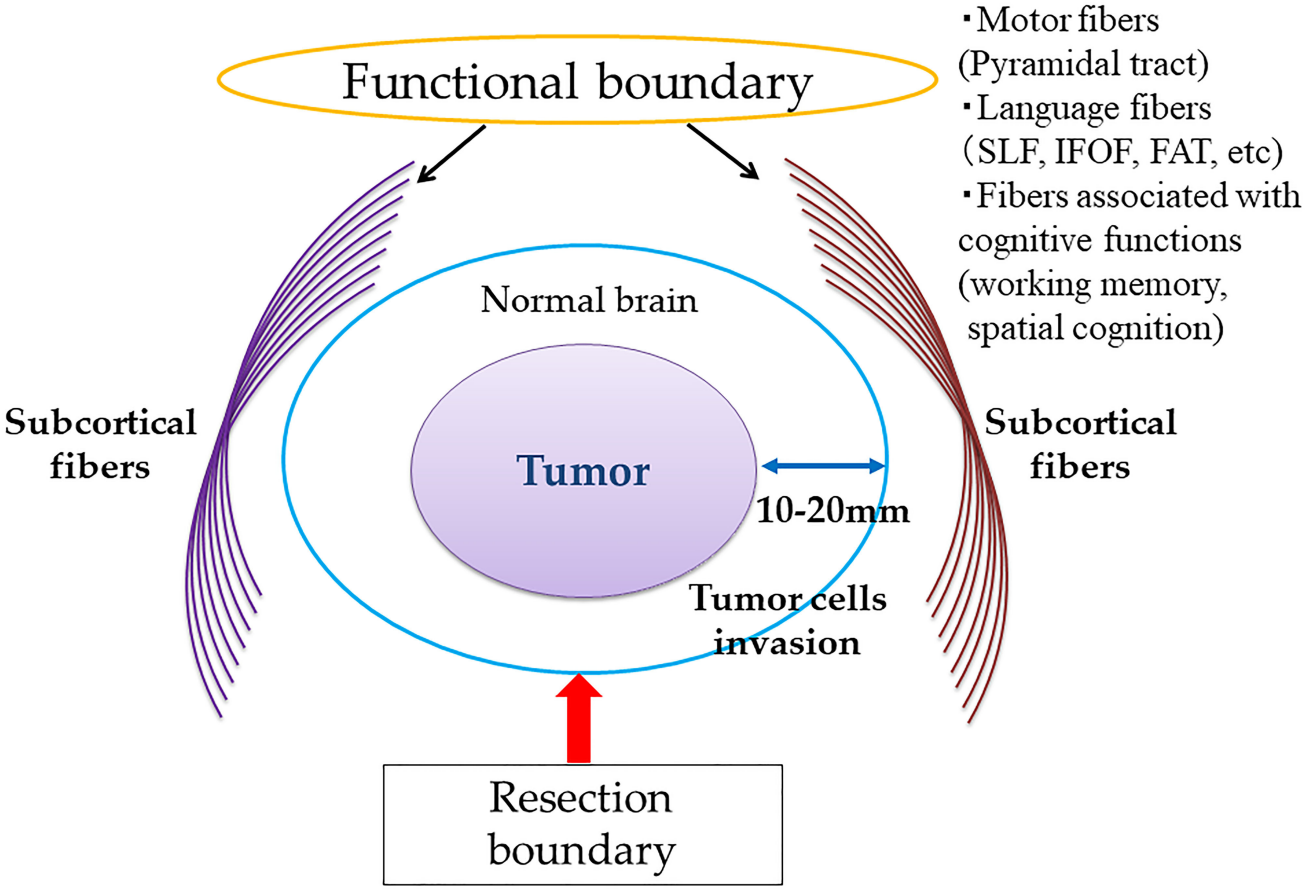
Conceptual diagram of supratotal resection of diffuse gliomas with awake brain mapping.

A clinical study by Duffau et al. reported that supratotal resection may improve the clinical outcomes of patients with WHO grade II gliomas who underwent awake brain surgery after a long-term period, of a mean follow-up period of 11 years (20). This study demonstrates that supratotal resection, extending beyond the abnormalities detected by T2- or FLAIR-weighted MRI, provides a survival benefit, as tumor cells may be present at a distance of 10–20 mm from the tumor boundaries on MRI (54). As a greater EOR of tumors, including gross total or supratotal removal, could increase PES and OS in patients with WHO grade II gliomas, many neurosurgeons worldwide have recently attempted to perform supratotal resection of tumors whenever possible with the use of awake functional mapping. A conceptual overview of supratotal resection for diffuse gliomas with awake functional mapping is shown in Figure 2. To achieve margin removal around MRI-detected abnormalities, performing classical motor and/or language mapping throughout surgical resection is recommended. Furthermore, to evaluate working memory and spatial cognition, stimulation of the fibers associated with cognitive functions has been proposed.

### Supratotal Resection and Tumor Location in the Dominant or Non-dominant Hemispheres

When the tumor is located in the non-dominant hemisphere, such as in the frontal and temporal poles, or inside the occipital lobes of patients who already show visual deficits before surgery, supratotal resection via lobectomy can be performed without difficulty. Shah et al. reported that lobectomy for GBM of the non-dominant hemisphere significantly improved PFS and OS, compared to the GTR group (55). Moreover, the authors found no increase in postoperative complications in these patients and concluded that supratotal resection is the best tailored technique for small lesions arising in the non-dominant frontal, temporal, and occipital poles.

In contrast, if the tumor is within the dominant hemisphere for motor and language functions, supratotal resection and gross total removal cannot be accomplished. Yordanova et al. attempted to perform supratotal resection of WHO grade II gliomas within non-eloquent areas of the left language-dominant hemisphere, and reported that four out of 15 patients experienced tumor recurrence without anaplastic transformation, with a mean postoperative follow-up duration of 35.7 months (21). The authors emphasized that it is crucial to delay anaplastic transformation and delay the use of adjuvant therapy, such as chemoradiotherapy, by supratotal resection.

### Supratotal Resection and Subcortical Fibers

**Figure 3:**
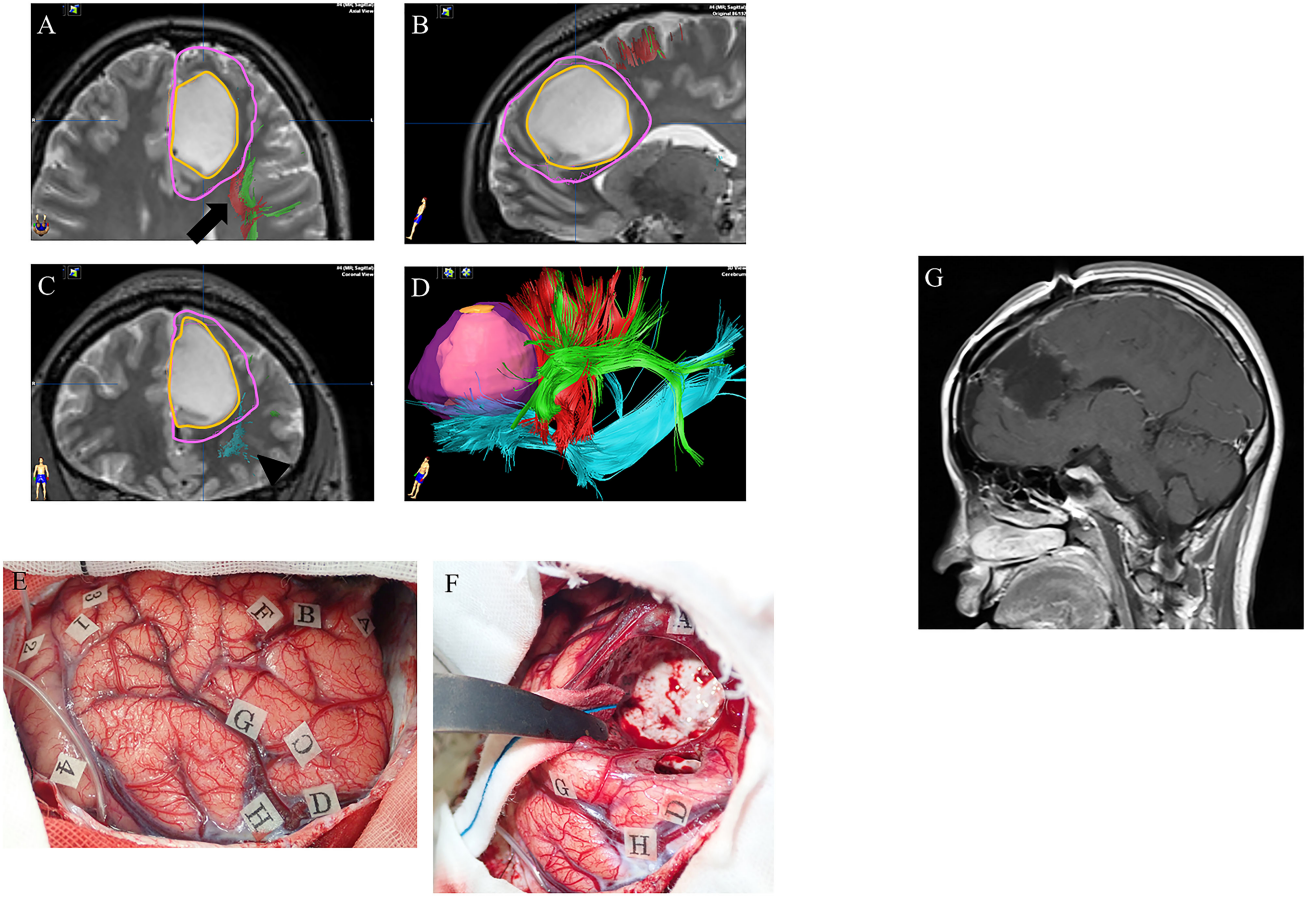
A case of left frontal lower-grade glioma in a 30-year-old right-handed female with no relevant medical history. Preoperative axial **(A)**, sagittal **(B)**, and coronal **(C)** T2-weighted MRI show a high intensity mass in the left frontal lobe. Preoperative three-dimensional tractography **(D)** shows the tumor itself (orange) and the planned resection area (violet; supratotal resection) surrounded by FAT (red; arrow in panel **A**), SLF (green; arrow in panel **A**) and IFOF (blue; arrowhead in panel **C**). Red, FAT; green, SLF; blue, IFOF; FAT, frontal aslant tract; SLF, superior longitudinal fasciculus; IFOF, fronto-occipital fasciculus. Intraoperative photograph prior to resection **(E)**, showing letter tags that indicate tumor boundaries (A–D) and the planned resection area for supratotal resection (E–H). Stimulation over the precentral gyrus induced speech arrest (number tags: 2 and 4), as did stimulation over the opercular part of the inferior frontal gyrus (number tags: 1 and 3). **(F)** Intraoperative photograph obtained after supratotal resection. Stimulation over the IFOF induced semantic paraphasia (number tag: 31), and stimulation over the FAT induced speech arrest (number tags: 33, 34). FAT, frontal anterior tract; IFOF, fronto-occipital fasciculus. Postoperative sagittal T1-weighted MRI with gadolinium enhancement **(G)** showing supratotal resection with awake brain mapping.

Our research group assessed the efficacy of awake functional mapping for supratotal resection of frontal WHO grade II and grade III gliomas in both the dominant and non-dominant hemispheres (53). In total, 11 patients with diffuse frontal WHO grade II and grade III gliomas who underwent awake brain surgery and supratotal resection were analyzed. The oral and written language functions of the tumors in the language-dominant frontal lobe were preserved using counting and picture-naming tasks. Using such intraoperative language tasks, awake functional mapping can detect anatomy-functional associations, which has revealed the “language connectivity” in the human brain. In language-associated subcortical fibers in the frontal lobe, the IFOF is one of the longest association fiber tracts, which constructs a ventral pathway that passes through the deep areas of the temporal lobe and insula, connecting the occipital cortex and posterior temporo-occipitoparietal regions to the orbitofrontal, prefrontal, and dorsolateral prefrontal areas (56). This ventral pathway is related to semantic processing, and stimulation of the IFOF during awake surgery using picture-naming tasks leads to semantic paraphasia (57). Furthermore, the superior longitudinal fasciculus (SLF) connects the inferior frontal gyrus (IFG) and ventral premotor cortex to temporoparietal language regions, which construct a dorsal pathway in the left frontal lobe (58). During awake surgery, phonemic paraphasia and articulatory disorders are elicited during direct stimulation. The frontal aslant tract (FAT), which was discovered only in the last decade, connects the pre-supplementary motor area (pre-SMA) and SMA in the medial frontal areas of the superior frontal gyrus (SFG), and posterior inferior frontal gyrus, which is part of Broca's area in the frontal lobe (59). The FAT is believed to play a role in self-initiated speech, involving the pre-SMA, SMA, and IFG. Our group performed resection extending to the functional boundaries, defined by the white matter fibers FAT, IFOF, and SLF (Figure 3), to perform extended resection of a tumor margin beyond MRI abnormalities in left frontal WHO grade II and grade III gliomas.

**Figure 4:**
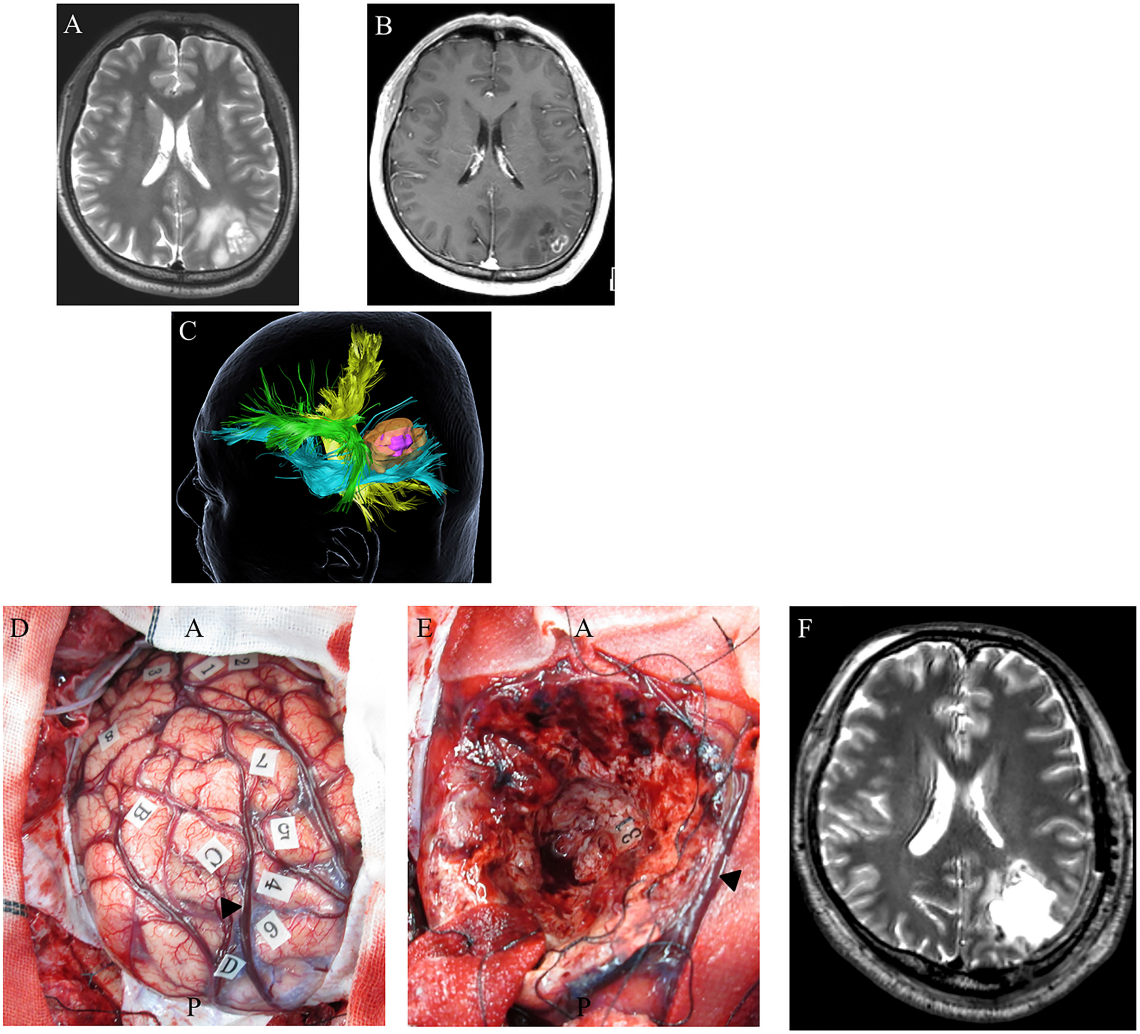
A 48-year-old right-handed male with *IDH*-wildtype glioblastoma (GBM). Preoperative axial T2-weighted **(A)** and axial T1-weighted MRI with gadolinium enhancement **(B)**, showing a high-intensity abnormal area in the left inferior parietal lobule including an enhancing region at the superficial area. Preoperative three-dimensional tractography **(C)** showing a yellow-colored fiber tract bundle showing the corticospinal tract. The green area shows the superior longitudinal fasciculus, the blue fiber tract bundle shows the inferior fronto-occipital fasciculus, and the high-intensity area on T2-weighted MRI is highlighted in orange. The violet-colored lesion in the orange-colored area showed an enhancing mass. **(D)** Intraoperative photograph obtained before tumor resection, with letter tags indicating tumor boundaries **(A–D)**. Stimulation over the postcentral gyrus induced convulsions around the mouth (number tags: 1, 2); stimulation over the left superior parietal lobule induced cessation of right upper limb movement (number tags: 4–7); arrowhead: intraparietal sulcus. **(E)** Intraoperative photograph obtained after supratotal resection. Stimulation over the IFOF induced semantic paraphasia (number tag: 31). Postoperative axial T2-weighted MRI **(F)** revealed no tumor following supratotal resection.

In contrast, the SLF/arcuate fasciculus complex and the IFOF were mainly associated with parietal tumors when attempting to achieve supratotal resection (Figure 4) (60). Subcortical stimulation of the SLF/arcuate fasciculus complex induces disturbances in spatial attention. Furthermore, rightward deviation was generated using a line bisection test. In the deep area of the parietal lobe, semantic paraphasia was induced by stimulating the deep side of the tumor cavity, which was caused by the stimulation of the IFOF.

During awake brain mapping of the temporal tumor for supratotal resection, direct electrical stimulation detects the IFOF, temporal part of the SLF, inferior longitudinal fascicle, and optic radiation. When the IFOF is stimulated, semantic paraphasia is also induced. Phonological disorders are elicited when the temporal SLF subserves the dorsal stream of the brain. The detection of the inferior longitudinal fascicle generates reading disturbances during stimulation. Optic radiation, which is deeply located, induces transient visual disturbances via stimulation.

### Role of Supratotal Resection for Glioblastoma

Several studies have reported that a greater EOR, such as that achieved by supratotal resection, might improve the survival of patients with GBM by decreasing postoperative neurological deficits, while preserving white matter fibers associated with motor, language, and neurocognitive functions (60–66) (Figure 4). A systematic review of several clinical studies on the supratotal resection of GBM was therefore conducted (Table 1).

**Table 1:** Supratotal resection of glioblastomas (literature review).

	**Number of patients**	**Definition of supratotal resection**	**Number and rate of supratotal resection cases (%)**	**OS**	**PFS**	**Adjuvant therapy**
Eyüpoglu et al. (61)	105	Beyond obvious contrast enhancement using 5-ALA and iMRI	30 (29%)	18.5 vs. 14 months (vs. GTR)	NA	RT + TMZ
Li et al. (62)	643	Resection over 53.21% of FLAIR	159 (25%)	20.7 vs. 15.5 months (vs. <53.21% of FLAIR)	NA	NA
Esquenazi et al. (63)	86	Beyond the zone of enhancement using subpial technique	25 (29%)	54 vs. 16.5 months (vs. GTR)	NA	RT + TMZ ± BCNU wafer
Grossmann et al. (66)	103	≤ 46% of remnant FLAIR (3 months post operation)	NA	26.7 vs. 13.4 months (vs. GTR)	NA	RT + TMZ
Pessina et al. [65)	282	Resection 100% of FLAIR	21 (7%)	28.6 ± 5.2 vs. 16.2 ± 1.2 months (vs. GTR)	24.5 ± 2.4 vs. 11.9 ± 0.6 months (vs. GTR)	TMZ + RT, 6-8 cycles TMZ
Glenn et al. (64)	32	Removal of at least 1 cm of brain tissue surrounding the enhancement	7 (21.9%)	24 vs. 11 months (vs. GTR)	15 vs. 7 months (vs. GTR)	NA

*OS, overall survival; PFS, progression free survival; GTR, gross total resection; TMZ, temozolomide; RT, radiotherapy; NA, not available; BCNU, carmustine; 5-ALA. aminolevulinic acid; iMRI, intraoperative magnetic resonance imaging; FLAIR, fluid-attenuated inversion recovery; DC, dendritic cell*.

Eyüpoglo et al. evaluated the role of supra-complete resection surgery using a dual intraoperative visualization approach combining intraoperative MRI with neuro-navigation and 5-ALA in GBM (61). In this study, 75 patients underwent gross total resection using intraoperative MRI and navigation. Thirty patients underwent supratotal resection using a surgical strategy with 5-aminolevulinic acid (5-ALA) and intraoperative MRI. The median OS of patients treated according to the surgical strategy using intraoperative MRI with navigation was 14 months, whereas those who underwent surgery with 5-ALA and intraoperative MRI had a significantly longer median survival time of 18.5 months. Thus, it was concluded that supra-complete glioma resection leads to significantly prolonged OS in patients with GBM.

Li et al. (62) assessed the role of supratotal resection in 876 GBM patients. In this study, supratotal resection was defined as complete resection of the T1-enhancing regions with a small portion, with additional resection of the surrounding fluid-attenuated inversion recovery (FLAIR) abnormality. The authors showed that resection of ≥ 53.21% of the surrounding FLAIR abnormality beyond contrast-enhanced areas was associated with a significant prolongation of survival, compared to less extensive tumor resection (median OS: 20.7 and 15.5 months, respectively). These results suggest that supratotal resection with the resection of the FLAIR abnormality region can lead to longer survival of patients with GBM, without increases in postoperative morbidity.

Esquenazi et al. retrospectively evaluated 86 patients with primary GBM with or without carmustine (BCNU) wafer implantation (63). They concluded that the effect of supratotal resection on OS surpassed the effects of tumor volume, age, and KPS score.

Pessina et al. evaluated the role of FLAIR abnormality removal in a retrospective study of 282 newly diagnosed GBM patients. They reported that the median OS for supratotal resection was 28.6 months, compared with a gross total resection OS of 16.2 months. Furthermore, fewer patients showed worsening neurological deficits after supratotal resection (4.8%) than after gross total (13.3%) or subtotal resection (5.6%). This large clinical study suggests that supratotal resection beyond contrast-enhanced boundaries could represent a promising strategy for improving the survival of GBM patients (65).

Glenn et al. reported that patients undergoing supratotal resection for temporal lobe GBM showed a significantly improved median PFS (15 months) compared to those who underwent gross total resection (7 months) (64). Moreover, a median OS advantage was also found in the supratotal resection group at 24 months compared to the gross total resection group at 11 months.

### Role of Supratotal Resection in the Treatment of WHO Grade II Gliomas

The first reports on supratotal resection of WHO grade II gliomas were published by Yordanova and Duffau et al. (21) (Table 2). Fifteen right-handed patients with 17 tumors underwent the resection of WHO grade II gliomas using intraoperative awake brain mapping. In this series, total resection was accomplished for all 17 tumors, whereas supratotal resection was performed for 15 tumors. Only four of the 15 patients in whom supratotal resection was achieved experienced tumor recurrence without malignant transformation, and the mean time to tumor recurrence was 38 months. The recurrence rate in the control group was 41%, whereas that in the supratotal resection group was 26%.

**Table 2:** Supratotal resection of WHO grade II gliomas (literature review).

	**Number of patients**	**Tumor classification (WHO grading)**	**Definition of supratotal resection**	**Number of supratotal resection case (%)**	**Adjuvant therapy**	**Clinical outcome**	**Mean follow-up duration and outcomes**
Yordanova et al. (21)	15	WHO grade II glioma	Resection extending beyond the area of MRI signal abnormalities	15 (100%)	None after the surgery (1 patient received radiotherapy at the relapse 6 years after the surgery)	Adjuvant treatment, anaplastic transformation, KPS, postop seizures, recurrence rate	35.7 months Average time to recurrence: 38 months
Duffau et al. (67)	11	WHO grade II glioma	A margin of parenchyma was removed around the preoperative FLAIR-weighted signal abnormality with a larger volume of the surgical cavity as compared with the presurgical tumor volume	3 (27.2%)	None after the surgery	Adjuvant treatment, KPS, postop seizures	40.0 months No anaplastic transformation
Duffau et al. (20)	16	WHO grade II glioma	A complete removal of any signal abnormalities with a volume of the postoperative cavity larger than the preoperative tumor volume	16 (100%)	None after the surgery, chemotherapy (*n* = 3), radiotherapy (*n* = 2)	Adjuvant treatment, KPS, malignant transformation, postop seizures, relapse time	132 months Average time to relapse: 70.3 months
Lima et al. (69)	21	WHO grade II glioma	A margin of parenchyma was removed around the preoperative FLAIR or T2-weighted sequence signal abnormality, with a larger volume of the surgical cavity as compared with the presurgical tumor volume	4 (19.0%)	Chemotherapy (*n* = 6), radiotherapy (*n* = 1)	Adjuvant treatment, KPS, postop seizures, tumor regrowth	49 months Alive, enjoy a normal life
Lima et al. (68)	19	WHO grade II glioma	A margin of parenchyma was removed around the preoperative FLAIR or T2-weighted sequence signal abnormality, with a larger volume of the surgical cavity as compared with the presurgical tumor volume	5 (26.3%)	Chemotherapy (*n* = 5), radiotherapy (*n* = 2)	Adjuvant treatment, KPS, postop seizures	62.4 months PFS: not reached

*WHO, World Health Organization; KPS, Karnofsky Performance Status; NA, not available; MRI, magnetic resonance imaging; FLAIR, fluid-attenuated inversion recovery*.

Duffau et al. analyzed 11 asymptomatic patients with WHO grade II gliomas in the language-dominant hemisphere (67). Supratotal resection was achieved in three patients by intraoperative awake mapping. No mortality or permanent postoperative neurological deficits were observed in any patient. Furthermore, no anaplastic transformation was observed in these cases, with a mean follow-up period of 40 months after tumor resection.

Lima et al. reported the efficacy of supratotal resection based on functional boundaries for incidental WHO grade II gliomas in a group of 19 patients (68, 69). Supratotal resection was achieved in five patients in this study. The PFS rate was significantly higher in patients who underwent total or supratotal resection than in those who underwent partial or subtotal resection (not reached vs. 65 months, respectively). Six months after surgery, all 19 patients were free of permanent neurological disturbances, and 18 had a postoperative KPS of 100.

### Role of Supratotal Resection in the Treatment of WHO Grade III Gliomas

Rossi et al. assessed the association between supratotal resection and 319 *IDH*-mutated WHO grade II and grade III gliomas (70) (Table 3). This large retrospective study evaluated the relationship between supratotal resection and survival, including PEF and OS, in a series of WHO grade II and grade III gliomas. They showed that PFS was significantly longer in patients with *IDH*-mutated WHO grade II and grade III gliomas, including astrocytomas and oligodendrogliomas, who achieved supratotal resection than in those who underwent gross total resection. Furthermore, supratotal resection was significantly associated with a reduced rate of better OS and malignant transformation (28, 71).

**Table 3:** Supratotal resection of WHO grade III gliomas (literature review).

	**Number of patients**	**Tumor classification (WHO grading)**	**Definition of supratotal resection**	**Number of supratotal resection case (%)**	**Adjuvant therapy**	**Clinical outcome**	**Mean follow-up duration and outcomes**
Motomura et al. (53)	9	WHO grade II glioma, WHO grade III glioma	Tumor resection extending beyond the abnormal MRI-verified area, which indicated that the volume of the postoperative cavity was larger than the preoperative tumor volume	9 (100%)	NA	WMS-R, SLTA, FAB, WAIS-III	NA NA
Rossi et al. (70)	319	WHO grade II glioma, WHO grade III glioma	Complete removal of any signal abnormalities, with the volume of the postoperative cavity larger than preoperative tumor volume	110 (35%)	Chemoradiotherapy (17.6%), chemotherapy only (41.2%)	Adjuvant treatment, anaplastic transformation, postop seizures, recurrence rate, OS, PFS, MPFS	6.8 years PFS at 92 months; 94%

*WHO, World Health Organization; WMS-R; Wechsler memory scale revised, SLTA; Standard Language Test of Aphasia, FAB; Frontal Assessment Battery, WAIS-III; the third version of Wechsler adult intelligence score; OS, overall survival; PFS, progression free survival; MPFS, malignant progression free survival, NA, not available*.

Motomura et al. found that an EOR threshold of 85.3% was beneficial for the treatment of WHO grade II and grade III gliomas. Improved PFS among patients with WHO grade II and grade III gliomas is predicted by a greater EOR (> 85.3%) as a result of aiming for supratotal or gross total resection (72). Their findings also indicated that an EOR of ≥ 100%, including supratotal resection, was significantly correlated with a favorable PFS in patients with WHO grade II and grade III gliomas. Based on these results, maximal safe tumor resection can be considered an important prognostic factor for improving the survival of patients with WHO grade II and grade III gliomas. Moreover, this study also emphasized the efficacy of awake mapping for supratotal resection of frontal WHO grade II and grade III gliomas while preserving motor, language, and neurocognitive functions (53).

### Present Issues in Supratotal Resection

In GBM, supratotal resection prolongs OS by 18.5–54 months and PFS by 15–24.5 months (61–66) (Table 1). In contrast, several studies have reported that in WHO grade II gliomas, supratotal resection provides a significant increase in PFS with no anaplastic transformation and prolongs the average time to recurrence (20, 21, 67–69) (Table 2). Regarding WHO grade II and III gliomas (i.e., lower-grade gliomas), OS, PFS, and malignant progression-free survival (MPFS) were significantly prolonged in a large retrospective study (70). However, several issues to consider regarding the efficacy of supratotal resection of gliomas. First, the definition of supratotal resection remains ambiguous. Although some studies have defined supratotal resection as resection extending beyond the area of MRI signal abnormalities, the extent to which the tumor needs to be resected to achieve supratotal resection and the percentages of tumor resection that are associated with better survival of gliomas is unclear. Second, the tumor location in relation to the eloquent area is a confounder in terms of resectability. In cases where the tumor is within the dominant hemisphere for neurological function, supratotal resection cannot be achieved. Thid, it is essential to consider tumor malignancy during supratotal resection. Tumors with biologically less malignancy may be more amenable to supratotal resection because the cells of such tumors develop relatively locally compared to highly malignant gliomas. Finally, there may be potential for significant bias when selecting only patients eligible for supratotal resection. Such cases feature a relatively small tumor size and location in the non-eloquent area.

## Conclusions

Despite aggressive treatment of GBM and WHO grade II and grade III gliomas, patients are still at a risk of tumor recurrence. Supratotal resection may improve the survival of patients with GBM and WHO grade II and grade III gliomas without increasing the risk of postoperative neurological deficits, while preserving some subcortical fibers associated with motor, language, and neurocognitive functions. Although several studies have provided novel information regarding the effect of supratotal resection with awake brain mapping on the survival of patients with gliomas, their results are limited compared to those of prospective clinical trials, as retrospective studies may be influenced by unrecognized biases. Therefore, a larger prospective study involving multiple independent research centers is required to establish the role of supratotal resection in awake brain surgery.

## Author Contributions

KM and RS: experimental design. FO and KA: collection and assembly of data. KM, FO, KA, and RS: analysis and interpretation of the data. KM: manuscript writing. All authors contributed to the article and approved the submitted version.

## Funding

This work was supported by a Grant-in-Aid for Scientific Research (C) awarded to KM (No. 21K09174) from the Japan Society for the Promotion of Science (JSPS).

## Conflict of Interest

The authors declare that the research was conducted in the absence of any commercial or financial relationships that could be construed as a potential conflict of interest.

## Publisher's Note

All claims expressed in this article are solely those of the authors and do not necessarily represent those of their affiliated organizations, or those of the publisher, the editors and the reviewers. Any product that may be evaluated in this article, or claim that may be made by its manufacturer, is not guaranteed or endorsed by the publisher.
